# Congenital Spigelian Hernia Combined with Bilateral Inguinal Hernias

**DOI:** 10.4274/balkanmedj.2017.1306

**Published:** 2018-09-21

**Authors:** Xenophon Sinopidis, Antonios Panagidis, Vasileios Alexopoulos, Ageliki Karatza, George Georgiou

**Affiliations:** 1 Department of Pediatric Surgery, University of Patras School of Medicine, Patras University Hospital, Patras, Greece; 2 Department of Pediatric Surgery, Karamandaneion Children’s Hospital, Patras, Greece; 3 Department of Pediatrics, University of Patras School of Medicine, Patras University Hospital, Patras, Greece

This report aims to point out the uncommon presentation of a Spigelian hernia during infancy, which is considered in general as an adulthood problem. A 32-week-of-gestation male with a birth weight of 1900 g presented with bilateral inguinal hernias. A third hernia was located at the lateral edge of the rectus abdominis muscle above the left inguinal hernia. Ultrasonography revealed preperitoneal fat protruding through a defect of the transversalis fascia (Figure 1a). Due to immaturity-related respiratory distress, surgical correction of the inguinal hernias was performed in the second month. Correction of the third hernia was performed in the fifth month, deciding to avoid the prolongation of anesthesia time and the operative stress and implementing a conservative approach to a situation that has never been confronted before (Figure 1b).

A hernial sac containing the small intestine was identified intraoperatively, laterally to the rectus abdominis muscle, below the arcuate line, and protruding through a defect of the transversalis fascia and the transversus abdominis muscle. Preperitoneal fat adhered to the tip of the hernial sac was the first to be recognized (Figure 1c). Dissection, inspection, and ligation of the sac were followed by closure of the fascia and the muscle gap. The diagnosis was a Spigelian hernia. Postoperative follow-up until the age of 18 months was uneventful. Written informed consent was obtained from the patient's parents.

A Spigelian hernia is defined as a hernia of the Spigelian fascia, the part of the transversus abdominis avponeurosis extending from the semilunar line to the lateral edge of the rectus abdominis (1,2). Pediatric Spigelian hernia of nontraumatic etiology has been described in 71 sporadic cases (age 0-18 years) in the English literature (1). The largest published series included only 8 children (1).

Infantile Spigelian hernia is considered as congenital in nature. It is related to an intrinsic structural predisposition of the anterior abdominal wall (3). Some authors have suggested a theory that the perforating vessels may weaken the fascia, enabling the entrance of fat and hernia formation (4).

The association of Spigelian hernia with undescended testes created the hypothesis of a distinct clinical syndrome with a common pathogenetic origin of both pathologies (1,2,5); embryonic testis descent in between the layers of the anterior wall results in testicular ectopia and a secondary Spigelian hernia (5).

The anatomic vicinity of the structures of the inguinal canal, Hesselbach’s triangle, and the Spigelian zone in the small-sized infant render possible that a Spigelian hernia may be misdiagnosed as inguinal (3). The rate of strangulation has been considered to be high, estimated in 20% of operated cases (3).

An early presentation in the postnatal life, a high incarceration rate, and the diagnostic difficulty of uncomplicated cases lead to infantile morbidity. The possibility of a Spigelian hernia should be included in the clinical examination of the abdominal wall and not omitted as a disease of late adulthood. Informed consent was obtained by the parents of the infant.

## Figures and Tables

**Figure 1:**
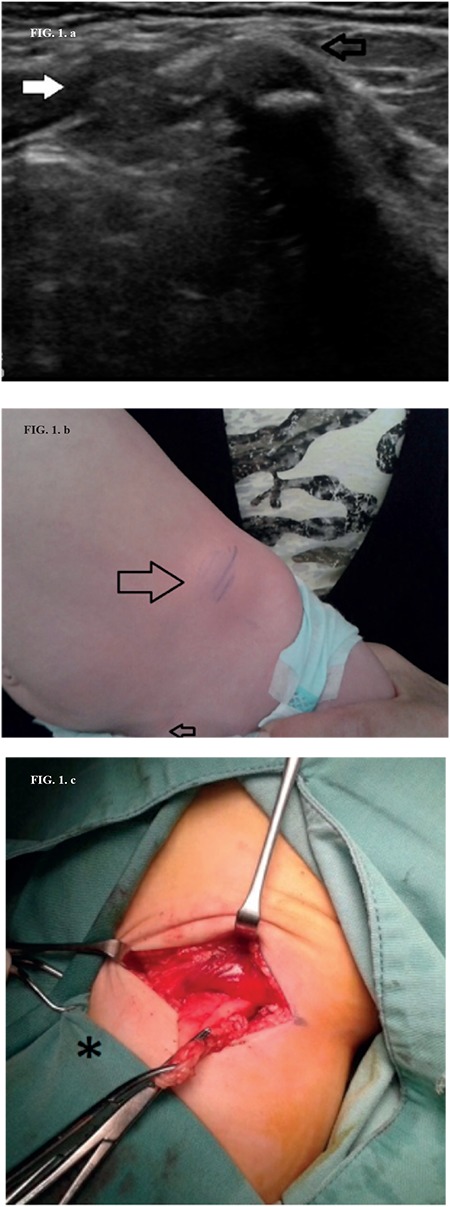
Ultrasound assay: Preperitoneal fat and bowel herniating through the transversalis fascia (white arrow). Iliac crest (black arrow) (a). Preoperative presentation of the Spigelian hernia (large arrow). Left inguinal hernia operative scar (small arrow) (b). Preperitoneal fat and peritoneum herniating through the transversalis fascia. Left inguinal incision site (asterisk) (c).
